# Adopting an Extended Theory of Planned Behaviour to Examine Buying Intention and Behaviour of Nutrition-Labelled Menu for Healthy Food Choices in Quick Service Restaurants: Does the Culture of Consumers Really Matter?

**DOI:** 10.3390/ijerph20054498

**Published:** 2023-03-03

**Authors:** Abu Elnasr E. Sobaih, Mohamed Algezawy, Ibrahim A. Elshaer

**Affiliations:** 1 Management Department, College of Business Administration, King Faisal University, Al-Ahsa 31982, Saudi Arabia; 2 Faculty of Tourism and Hotel Management, Helwan University, Cairo 12612, Egypt; 3 Faculty of Tourism and Hotel Management, Suez Canal University, Ismailia 41522, Egypt

**Keywords:** nutrition-labelled menu, healthy food choice, quick service restaurants (QSRs), cultural differences, health consciousness, theory of planned behaviour (TPB), intention to buy, intention to recommend

## Abstract

This research aims to examine an extended model of the Theory of Planned Behaviour (TPB) to understand the determinants of consumers’ intentions to buy and recommend nutrition-labelled menu (NLM) items for making healthy food choices. The research examines the influence of attitude towards behaviour (ATT), subjective norms (SNs), perceived behavioural control (PBC) and health consciousness on consumers’ intentions to buy and recommend NLM. The research also examines the role of culture in shaping buying and recommendation intentions of NLM by undertaking a comparative study of the extended model among consumers in two different countries that have enough variation based on Hofstede’s cultural dimensions, i.e., the Kingdom of Saudi Arabia (KSA) and the United Kingdom (UK). The results of questionnaire surveys analysed with SmartPLS version 4 showed that ATT, SNs and health consciousness significantly predict intentions to buy NLM items among KSA consumers in quick service restaurants (QSRs). However, PBC did not have a significant influence on KSA consumers’ intentions to buy NLM items. On the other hand, ATT, PBC and health consciousness significantly predict intentions to buy NLM items among UK consumers in QSRs. Nonetheless, SNs did not have a significant influence on UK consumers’ intentions to buy NLM items. The intention to buy NLM significantly predicts the intentions to recommend NLM among consumers in both countries (KSA and UK). The results of a multi-group analysis showed significant differences between the KSA and the UK regarding the influence of both SNs and PBC on consumers’ intentions to buy NLMs as well as on their indirect influence on intentions to recommend NLM items. The results value the role of culture in shaping consumers’ intentions to buy and to recommend NLM items for healthy food choices, which has numerous implications for international QSRs, policy makers, and academics.

## 1. Introduction

Quick service restaurants (QSRs) have been blamed for the introduction of junk and unhealthy food, which has contributed to abdominal and general obesity [1,2,3,4]. The increasing consumption of fast food provided by QSRs was found to be correlated with a high fat diet, a high Body Mass Index (BMI) and weight gain [5]. A recent report issued by the World Health Organisation (WHO) in June 2021 [6] showed that obesity has almost tripled since 1975. Additionally, the report showed that there are about 2 billion overweight adults and about a third of them are obese. Furthermore, rates of obesity among both adults and children are increasing, which contributes to the prevalence of several chronic diseases and health conditions. The report also confirmed that being overweight and obese kills more people than being underweight [6].

In order to manage the consumption of high calories and help consumers make healthier food choices, the governments of many countries, such as the USA and the UK, have forced food providers, e.g., restaurants, to adopt nutrition fact labels [7]. This was executed to help consumers identify the number of calories provided in each served meal. For example, in the USA, recent regulations under the Affordable Care Act, forced restaurants and retail food establishments to provide their consumers with calorie and other nutrition information for each served food item including food on display or self-served food [8]. In the UK, the government has asked all restaurants and cafes to provide calorie information on menus as a part of the government’s strategy to control obesity [9]. Several international QSRs, such as McDonald’s, have provided nutrition-labelled menus (NLM), to support consumers with nutrition information about each item in the menu. This nutrition information is provided at the website and at the point of service on display or on the placemat with each served food or drink item [10].

The growing interest of decision makers and many governments in managing nutrition and calorie labelling for promoting consumer healthy food choices has motivated scholars to examine the effect of nutrition information usage on consumers’ buying intentions and behaviour [7,11]. For example, a relationship between calorie labelling and total calories purchased has been found [12]. Additionally, the introduction of nutrition information and calories has contributed to a reduction in BMI and obesity [13]. However, “*the long-term effect of calorie labelling on fast-food purchases is unclear*” [10]. There is a paucity of research on the factors that make consumers use nutrition and calorie information to make a buying intentions or behaviour in QSRs [14]. Sobaih and Abdelaziz [14] called for further research, arguing that “*future researchers could examine this issue* [the culture] *by adopting an international comparative study between counties with different cultural dimensions to examine whether culture moderates customer choices of health foods*” (p. 13).

This research is a response to the call for more studies for understanding consumers’ buying intentions and behaviour of healthy food choice based on NLMs provided at QSRs, as recommended by recent studies from Petimar et al. [10] and Sobaih and Abdelaziz [14]. This research adopted an extended model by investigating the three determinants of the theory of planned behaviour (TPB) [15] along with health consciousness on consumers’ intentions to buy NLMs in QSRs for supporting healthy food choices. The research examines the influence of attitude towards behaviour (ATT), subjective norms (SNs), perceived behavioural control (PBC) and health consciousness (HC) on the intentions to make healthy food choices by considering the nutrition-labelled menu. The research also examines the role of culture on consumers’ intentions to buy and recommend healthy food through an extended model of TPB. More exactly, the research compares between consumers in two different countries, which have enough variation based on Hofstede’s cultural dimensions, i.e., the Kingdom of Saudi Arabia (KSA) and the United Kingdom (UK). Understanding the role of culture is important for international businesses when providing nutrition information to consumers in order to stimulate their intentions and make a decision of healthy food choices.

The following sections of the manuscript start with developing a research conceptual framework and building the research hypotheses (Section 2). Section 3 provides information about the research instrument, research population and sample as well as the data analysis technique adopted in the current research. Section 4 presents the results of the research structural model, its validity, and reliability. This section provides the results of two structural model in the KSA and the UK. Section 5 discusses the findings of the research and the results of two structural models. It compares the results with previous related studies and the implications of the study. Section 6 concludes the research and presents some ventures for future research.

## 2. Conceptual Framework and Hypotheses Development

### 2.1. The Influence of TPB Determinants and Health Consciousness on Consumers’ Intentions to Buy and to Recommend NLM Items

This research adopts a TPB framework to understand consumers’ intentions to buy and recommend NLM items for making healthy food choices in QSRs. The TPB framework is an expansion of the Theory of Reasoned Action (TRA) [16]. The TRA structure assumes that behavioural intention is the major antecedent of human behaviour. Furthermore, ATT and SNs are the two main antecedents and predictors of behavioural intention [16]. Attitude refers to an individual’s assessment of a given behaviour favourably or unfavourably, whereas SNs refers to the social influence that makes individuals engage or not in a given behaviour [15]. One of the main weaknesses of TRA is the assumption that individuals have full control regarding the behaviour they intend to practice; however, this may not always be true [15]. Hence, a new variable was added to the TRA called the PBC. The PBC refers to “*the perceived ease or difficulty of performing the behaviour*” ([15], p. 188). These three antecedents (ATT, SNs and PBC) of behavioural intention encompassed the three main determinants of behavioural intention in the TPB.

The TPB was adopted by several studies to examine consumers’ buying intentions and behaviour in various contexts. For instance, TPB was adopted to understand customers’ intentions to visit green hotels [17]. The results showed that ATT, SNs and PBC positively influence customers’ intentions to stay in a green hotel. Another study by Elshaer et al. [18] adopted TPB to examine customers’ intentions to generate food waste. The study showed that determinants of TPB (ATT, SNs and PBC) fully mediate the link between religiosity and food waste intention. Additionally, they partially mediate the link between the food consumption culture and the food waste intention. Shin et al., 2018 [19] examined customers’ intentions and behaviour regarding organic menus through the lens of TPB and a norm activation model. They found that ATT, SNs, PBC and personal norms are all determinants of customers’ intentions to buy organic menu items. Related to this, Shin et al., 2020 [20] found that ATT, SNs, and PBC significantly predict customers’ intentions regarding state-branded products. Additionally, consumers’ purchase intentions predict their actual buying behaviour of state-branded products. A recent study adopted TPB to examine customers’ intentions to buy nutrition-labelled items in fast food operations [14]. The results showed that only SNs and PBC significantly affect consumers’ intentions to buy NLM. However, there was no significant influence of ATT on customers’ behavioural intentions. The intention to buy NLM was found to significantly predict their visit to fast food operations that provide these NLMs and recommend them to others.

The founder of TPB, Ajzen [15], argued that other variables could be added to the TPB framework if they contribute to behavioural intention. Hence, several studies have added some variables to understand consumers’ behavioural intentions. For example, Shin et al. [20] added personal norms to the three constructs of TPB (ATT, SNs, PBC) to understand consumers’ intentions and behaviour towards organic menus. Like the study of Shin et al. [20], which added health consciousness for understanding customers’ intentions and behaviour regarding state-branded products, the current research extends TPB by adding health consciousness to assess consumers’ intentions to buy and recommend NLMs in QSRs. Health consciousness refers to the integration of health concerns in lifestyles [21]. While Shin et al. [20] found no significant influence of health consciousness on consumers’ intentions to purchase state-branded products. Yadav and Pathak [22] confirmed that health consciousness is a significant predictor of the intention to buy organic food. Other studies [23,24] confirmed a relationship between health consciousness and the intention to buy and consume local food items. This research will add to the body of academic literature and examine the effect of health consciousness on consumers’ intentions to buy NLMs.

**Figure 1:**
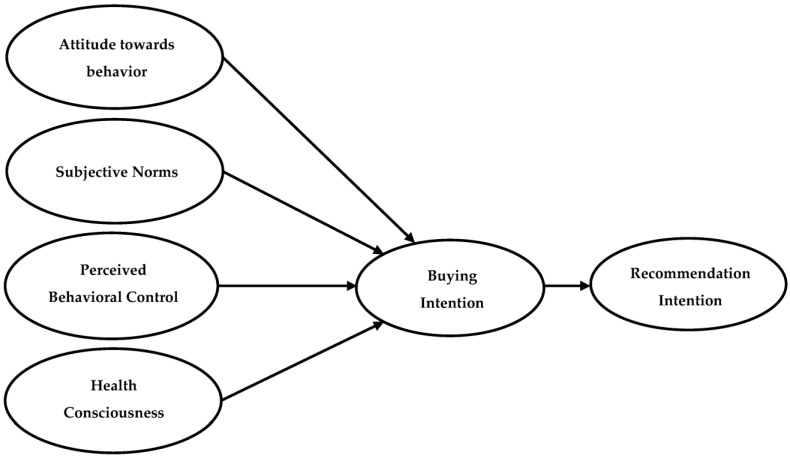
The research conceptual model.

Drawing on TPB and the above-discussed relationships, this research assumes that ATT, SNs, PBC and health consciousness significantly predict consumers’ intentions to buy MLM items for healthy food choices. Additionally, consumers’ intentions predict consumers’ intentions to recommend NLM items to others (see Figure 1). Therefore, hypotheses (H) 1–5 are proposed:

**H1:**  *Attitude significantly influences consumers’ intentions to buy NLMs in QSRs.*

**H2:**  *Subjective norms significantly influence consumers’ intentions to buy NLMs in QSRs.*

**H3:**  *Perceived behavioural control significantly influences consumers’ intentions to buy NLMs in QSRs.*

**H4:**  *Health consciousness significantly influences consumers’ intentions to buy NLMs in QSRs.*

**H5:**  *Consumers’ intentions to buy NLMs in QSRs significantly influence intentions to recommend NLMs to others.*

### 2.2. The Role of Culture

Hofstede [25] defined what causes individuals, groups, and communities to imitate certain attitudes and behaviours as “collective programming”. In that sense, every country has its own culture that drives the behaviours of its people. Recent research [26] on cultural differences among QSR consumers showed that culture plays a prime role in consumers’ perceptions of McDonald’s in four different countries (the US, Egypt, Vietnam and Malaysia) which drive variation in Hofstede’s cultural dimensions [25]. For instance, the research showed that “*the US perceived McDonald’s more critically than other countries whereas Egypt and Vietnam viewed it more favourably*” ([26], p. 391). The study of Lee and Ulgado [27] showed that international QSRs should consider cultural differences as customers have different expectations for the services based on their culture. For example, while American customers expect low prices in QSRs, Korean customers are focusing on service dimension factors such empathy. Furthermore, Qin et al. [28] confirmed that there is a variation in customer’s perceptions of QSRs based their culture. The study of Khan et al. [26] showed that American consumers are more concerned about the food quality in MacDonald’s than Vietnamese and Egyptian consumers. Additionally, Malaysian and Egyptian consumers value McDonald’s socially and emotionally whereas Americans see it as a place to consume food. Consumers with Eastern culture, e.g., Malaysian, value McDonald’s as a place for social gathering with family and friends while Americans perceive it as a place for convenience food.

There were several attempts by research to undertake cross-cultural studies for customers’ purchasing intentions and behaviours. Moon et al. [29] studied the effect of culture on buying personalized products online and found that individualism significantly influences purchase intentions. The study of Peña-García et al. [30] showed that national culture has a moderating role on consumers’ intentions and behaviours online. Another study by Sreen et al. [31] showed that collectivism as a dimension of culture significantly affects the three determinants of green purchase intentions (ATT, SNs, PBC). A study on repurchase intentions of fast food meals [32] showed the factors that affect repurchase intentions vary between Americans and Kuwaitis. For example, Kuwaitis often value non-food items such as staff attitude more than Americans who focus on food quality.

Based on these discussions, this research predicts significant differences in ATT, SNs, PBC, health consciousness and consumers’ intentions to buy and recommend NLMs in QSRs between two countries that have variation according to Hofstede’s cultural dimensions (e.g., the KSA and the UK) [25].

## 3. Methods

The approach adopted in the current study was a quantitative cross-sectional research design, where a thorough review of previous studies was carried out to extract the measures that suit the current study and contribute to the design of the theoretical framework. Based on this process, the study hypotheses were created. Subsequently, data were gathered by an instrument uploaded online and analysed employing PLS-SEM bootstrapping and a multi-group analysis method.

### 3.1. Sampling

The study targeted QSR consumers in the UK and the KSA. The survey was distributed through specialized data collection company in each targeted country. The role of the data collection company was to facilitate the process of data collection; however, the research team administered the whole process. Customers were contacted at the point of purchase, e.g., MacDonald’s, to voluntarily participate in the study. Only those who gave consent participated in the current study. To safeguard the privacy of participants, all data that could potentially reveal their identities were removed from the survey’s results. The contribution to fill in the survey was purely voluntary and anonymous. Consumers had the option of providing their name and age. A total of 900 surveys were distributed, with 450 in each country, and 400 valid responses were used for analysis in each country, with a response rate of 88%. The sample size of 400 responses in each country used in this study is appropriate for analysis using PLS-SEM. This satisfies the requirements set by Nunnally [33] of having at least ten responses per scale item (as the current study has 20 scale items, the minimum suggested sample size is 200). Additionally, it satisfies Hair et al.’s [34] standards of having at least 100–150 answers to achieve accurate estimates. According to Krejcie and Morgan’s [35] guidelines, when the population size is above 1,000,000, the minimal required sample size is 384 responses. Based on all these points, it can be determined that the existing sample size of 400 is sufficient for further analysis. The survey was made available in November and December 2022, and participants from each country were asked to provide their perceptions on the same set of questionnaire items.

### 3.2. Development of the Study Measures

Standard psychometric properties were employed to review the literature and choose the research measures. The variables of the theory of planned behaviour (TPB), including intention to buy and recommend, were assessed using a 17-item scale that was adopted from Ajzen [15] and Shin et al., 2019 [19]. This scale included items for attitude (4 items), subjective norms (3 items), perceived behavioural control (4 items), intention to buy (3 items) and intention to recommend (3 items). Additionally, health consciousness was operationalized using a 3-item scale adapted from Shin et al., 2020 [20]. The full measure adopted in the current study is presented in Appendix A. To increase the response rate [34], the items were pre-tested, and the questionnaire was kept as brief as possible. Only key demographic information was collected.

A specialized data collection company was utilized to obtain a high response rate. Consumers were invited to show their agreement on a 5-point Likert scale, going from “strongly disagree” (1) to “strongly agree” (5), instead of 7-point or 10-point Likert scales. The use of 5-point scales requires less time, is easy to answer [36] and enables the use of advanced multivariate statistical analysis methods [37].

As this study used a self-reported online survey, there is a possibility of common method variance (CMV) [33]. To address this potential issue, three techniques were adopted: (1) The dependent questions (intention to buy and intention to recommend) were placed before the independent questions (TPB and health consciousness items) in the survey; (2) Respondents’ private data were retained as confidential; (3) Harman’s single-factor method was used. This method involves subjecting all questions to exploratory factor analysis (EFA) in the SPSS program, with the constraint that just one factor will be retrieved with no rotating of the data. The results of the investigation showed that CMV was not an issue at any point throughout the inquiry, as just one variable explained about 36% (<0.50) of the variance in the data [34,38].

### 3.3. Methods of Data Analysis

The data analysis was performed in four successive stages. The respondents’ characteristics were described in the first stage. The second stage aimed to evaluate the research measurement psychometric properties such as reliability and validity. To achieve this, various statistical techniques were employed including Cronbach’s alpha (α), composite reliability (CR), cross loadings and average variance extracted (AVE). The third stage aimed to test the research model and hypotheses using a partial least squares structural equation modelling (PLS-SEM) approach. Finally, in the last phase (stage four), a multi-group analysis method was tested in PLS-SEM to identify any variance between the UK’s and the KSA’s QSR consumers.

## 4. Results

### 4.1. Respondents’ Characteristics (Stage 1)

The demographic characteristics of the targeted consumers such as their age, gender, education degree and diet status were collected through optional questions in the designed survey. As to consumers in the KSA, most respondents, i.e., 65%, were in the age range from 21 to <30 years, followed by those between 30 and <40 years, or 25%. Additionally, 7% of the respondents were under 21 years and only 3% were between the ages of 40 and 50, indicating that young consumers (usually 40 years or less) are the dominant segment of QSR customers in the KSA (90%). The sample had a similar proportion of female (52%) and male (48%) consumers. With respect to education levels, most the sample had completed a bachelor’s degree (60%) followed by those who had completed a doctoral or master’s degree (15%); 25% were secondary school consumers. In relation to diet condition, only 20% of the consumers were adopting a particular diet program; most were not (80%).

In regard to consumers in the United Kingdom, the majority of respondents in this country, 55%, were in the age range between 30 and <40 years followed by those aged between 40 and 50 (30%); 10% of consumers were aged from 21 to <30 years and only 5% of the consumers were <21 years. This shows that 85% of consumers of QSRs in the UK are adults (30 to 50 years old) and are the dominant segment of QSR customers in the UK. The sample had a similar proportion of female (51%) and male (49%) consumers. In relation to their education level, most the sample had completed a bachelor’s degree (66%) followed by those who had completed a doctoral or master’s degree (20%); 14% were secondary school consumers. Concerning diet condition, 40% of the consumers were following a particular diet program but most were not (60%).

### 4.2. Measurement Validity (Stage 2)

The research’s main construct was evaluated for convergent and discriminant validity using various methods such as factor loadings, composite reliability average variance extracted, Cronbach’s alpha, cross-loading, heterotrait–monotrait ratio of correlations and the Fornell–Larcker criterion before testing the proposed model and hypotheses. The factor loadings for all the measured items were calculated and screened to ensure they loaded on the appropriate construct, and all had values higher than the recommended threshold of 0.50 [39]. Table 1 illustrates that the factor loadings calculated for all the measured items in the two countries (the UK and the KSA) were between 0.737 and 0.949 and exceeded the suggested threshold of 0.50 [39].

Table 1 demonstrates that the values for Cronbach’s alpha and Composite Reliability (C.R) in the two groups of interest (the UK and the KSA) for which they were calculated were above the minimum accepted value in similar business research (>0.7) [40]. Additionally, the Average Variance Extracted (AVE) values were computed and compared to the minimum threshold value of 0.50 recommended by Hair et al. [39]. As shown in Table 1, the AVE values for all research constructs surpassed this threshold which approves the scale convergent validity in both countries (the UK and the KSA).

The discriminant validity was also evaluated using three criteria: cross-loadings, Fornell–Larcker and the HTMT ratio. In cross-loadings, items are expected to load more strongly to their reflective factor than to any other factor in the scale. The Fornell–Larcker criteria state that the correlation coefficient between measured constructs must be lower than the square root value of AVE, and that the HTMT ratio requires the correlation coefficient between constructs to be lower than the recommended level of 0.85 [39]. Table 2 shows the cross-loading metric, where the item loadings are correlated more strongly to their predetermined factors than to any other factor (in both data sets for the UK and the KSA). 

Furthermore, the assessment of Fornell–Larcker (Table 3 and Table 4) shows that the value of all squared roots of AVEs, written in bold and located in the diagonal part of the discriminant validity table, are higher than the correlation coefficient between model constructs. Similarly, Table 3 and Table 4 also show that all the HTMT values were lower than the recommended level. Therefore, it can be concluded that the measurement convergent and discriminant validity in both data sets (the UK and the KSA) were achieved, and the collected data were suitable for structural model evaluation.

### 4.3. Structural Model Evaluation and Hypothesis Testing (Stage 3)

The third stage of the analysis included evaluating the structural model of the study using the Partial Least Squares Structural Equation Modelling (PLS-SEM) method. The study’s constructs were then subjected to Smart PLS V.4 software according to the proposed hypotheses, and a path analysis was conducted using the bootstrapping resampling method with 5000 repetitions. All hypotheses were evaluated using the path coefficient (β), and only those with *p* values ≤ 0.05 were considered significant.

**Figure 2:**
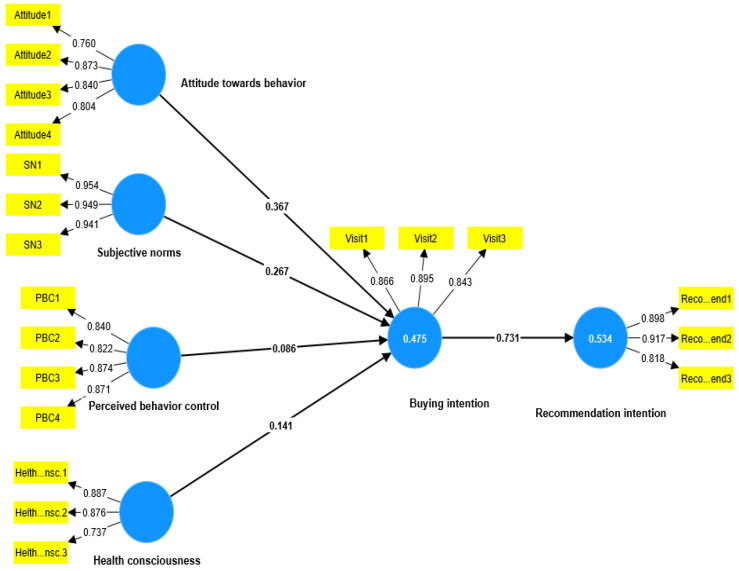
KSA research Model.

**Figure 3:**
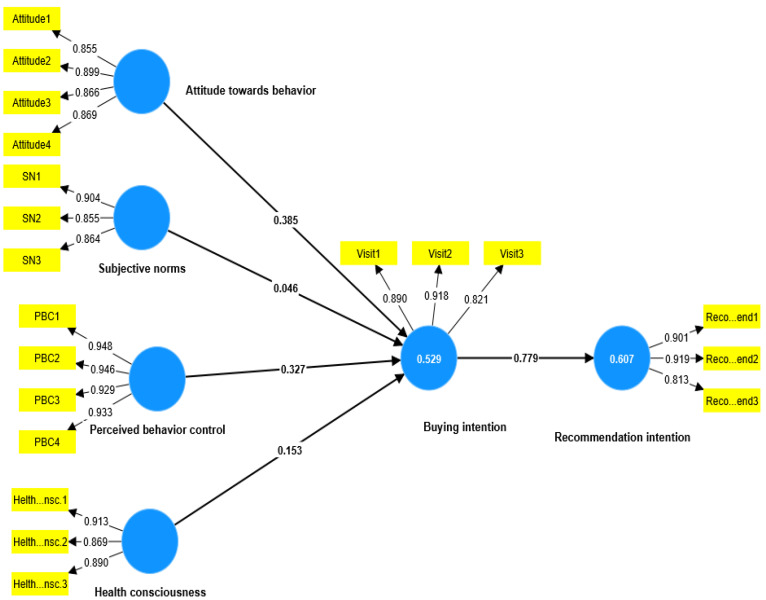
UK research model.

As shown in Figure 2 and Figure 3 and Table 5, the PLS-SEM results revealed that attitude towards behaviour has a positive influence on buying intention in both the data set for the KSA (β = 0.367, *t* = 7.530, *p* < 0.000) and for the UK (β = 0.385, *t* = 8.184, *p* < 0.000), which support H1 in both groups of interest. On the other hand, subjective norms in the KSA’s data set showed a positive significant influence on buying intention (β = 0.267, *t* = 5.385, *p* < 0.000). At the same time, it failed to significantly influence buying intention in the UK’s data set (β = 0.046, *t* = 1.226, *p* = 0.220), which means that H2 is supported in the KSA but not in the UK. Furthermore, perceived behaviour control failed to significantly influence buying intention in the KSA’s data set (β = 0.086, *t* = 1.281, *p* = 0.222). At the same time, perceived behaviour control succeeded in influencing buying intention with a significant *p* value in the UK’s data set (β = 0.327, *t* = 6.908, *p* < 0.000), which means that H3 is supported in the UK’s but not in the KSA’s data set. The positive significant effect of health consciousness on buying intention was slightly higher in the UK’s data set β = 0.153, *t* = 3.753, *p* < 0.000 than in the KSA’s data set β = 0.141, *t* = 3.084, *p* < 0.000, which supports H4 in both groups of interest. Similarly, the positive significant effect of buying intention on recommendation intention was slightly higher in the UK’s data set (β = 0.779, *t* = 24.832, *p* < 0.000) than in the KSA’s data set (β = 0.731, *t* = 20.746, *p* < 0.000), which supports H5 in both groups of interest.

In relation to the mediating impact of buying intention, the specific indirect effects in the PLS-SEM report were checked. They revealed that buying intention has a significant mediating impact between attitude towards behaviour and recommendation intention in the KSA (β = 0.269, *t* = 7.143, *p* < 0.000) and the UK (β = 0.300, *t* = 7.639, *p* < 0.000). Similarly, as shown in Table 5, buying intention succeeded in mediating the impact of perceived behaviour control and health consciousness.

On the other hand, buying intention failed to mediate the impact of subjective norms on recommendation intention in the UK’s data set (β = 0.036, *t* = 1.222, *p* = 0.222), while it succeeded in mediating the same relationships in the KSA’s data set (β = 0.165, *t* = 4.847, *p* < 0.000).

### 4.4. Multi-Group Analysis (Stage 4)

In the final stage (Stage 4), we examined the significant differences between the KSA and the UK in terms of the impact of attitude towards behaviour, subjective norms, perceived behaviour control and health consciousness on buying intention. We also checked the differences in the mediation analysis. The two groups’ models (the UK and the KSA) are compared to each other to find out the differences in the causal structure and therefore identify which path causes the variance between the two groups of interest. The findings showed that only two paths in the two groups of interest (the UK and the KSA) were the source of variance in the group analysis. The differences in path coefficients with their corresponding significant *p* value revealed that the effect of subjective norms on buying intention was stronger in the KSA than in the UK. Similarly, the differences in path coefficients and *p* value revealed that the impact of perceived behaviour control on buying intention was stronger in the UK than in the KSA. Furthermore, the mediating effects of buying intention on the relationship between subjective norms and recommendation intention, and between perceived behaviour control and recommendation intention, were found to be another two primary sources that can cause the variance between the two groups of interest. The findings of the multi-group analysis are presented in Table 6.

## 5. Discussion

This research has two main objectives. The first objective is to examine the extended model of TPB (Figure 1), which includes the influence of ATT, SNs, PBC and health consciousness on consumers’ intentions to buy and recommend NLMs in QSRs for healthy food choices. The second objective is to examine the effect of national culture on this extended model by undertaking a comparative study between two countries that have enough variation in Hofstede’s cultural dimensions. The results of structural model using Smart PLS v4 showed that ATT and SNs significantly predict intention to buy NLM items in QSRs among KSA consumers. These results are in line with the framework of TRA [16] and partially support TPB [15] in that only two determinants (ATT and SNs) affect the intentions of KSA consumers to buy NLM items. However, PBC did not have a significant influence on consumers’ intentions in the KSA to buy NLM items. This means that Saudi consumers did not find it easy to buy NLM despite their positive attitude and social influence. They do not have enough control on their behaviour, which could be due to lack of time or money [41]. On the other side, the results of UK model showed that both ATT and PBC were found to significantly predict the intention to buy NLM items in QSRs. This result partially supports TPB [15] in that two determinants (ATT and PBC) significantly influence behavioural intention. However, the results showed that SNs did not have a significant influence on consumers’ intention in the UK to buy NLM items. This could be because the UK has an individualist culture [25], hence their buying intentions are less likely to be influenced by their friends and peers.

Unlike the results of Shin et al. [20], which found that health consciousness did not affect consumers’ state-branded food purchase intentions and behaviours, the current study found a significant impact of health consciousness on intentions to buy NLMs to make healthy food choices among both KSA and UK consumers. These findings support previous research studies [22,23,24] where health consciousness significantly predicts consumers’ intentions to purchase organic menus or local food items. In addition, the results support the TPB framework [15] and previous research [14] where the intention to buy NLMs significantly predicts intention to recommend NLM among consumers in both countries (the KSA and the UK). Moreover, the results confirmed a mediating role for intention to buy NLM in the relationship between the three antecedents (ATT, SNs, PBC), health consciousness and the intention to recommend NLMs among consumers in the KSA and the UK. Nonetheless, the intention to buy NLM items has no mediation effect between SNs and recommendation intentions for NLM items among UK consumers.

The research adopted a multi-group analysis to investigate the role of culture in the extended model of TPB. Multi-group analysis shows whether there are significant differences between the KSA and the UK in the extended model of TPB, which affect consumers’ intentions to buy and recommend NLM items. The results of the multi-group analysis showed that the differences in culture between the two countries (the KSA and the UK) were significant in four relationships. First, the relationship between SNs and intention to buy NLM items has a significant difference between KSA and UK consumers. KSA consumers are more collective than UK consumers; hence, they are more affected by social influences than by individual societies, i.e., the UK. Second, the relationship between PBC and the intention to buy NLM items has a significant difference between KSA and UK consumers. UK consumers were found to have more control of their behaviour than KSA consumers. Third, SNs had an indirect effect on the intention to recommend NLMs through the intention to buy NLMs. The intention to buy NLMs has a mediating role between SNs and NLM items among KSA consumers with no mediation effect on UK consumers. Fourth, PBC has an indirect effect on the intention to recommend NLM through the intention to buy NLM. The intention to buy NLM has a mediating role between PBC and NLM items among UK consumers with no mediation effect on KSA consumers.

The above results contribute to the literature by adding health consciousness as a significant predictor and antecedent of consumers’ intentions to buy NLM items for healthy food choices. In addition, it has an indirect influence on consumers’ recommendation intentions of NLM items through buying intention. Hence, policy makers and international QSRs should pay high attention to promoting health consciousness among consumers to stimulate their intention to buy and recommend NLM items. The current research supports the work of Huang et al. [42] who stressed the role of policy makers in encouraging health consciousness to drive consumer’s healthy food choices. Policy makers should integrate health awareness about consumption of healthy food choices into the healthy lifestyle of consumers. They should undertake a health campaign to encourage consumers to use nutrition information and make healthy choices based on these NLMs provided to them. Legislation and regulations that enforce QSRs to implement NLMs are also important to encourage healthy food choices.

The results of this research confirm that international QSRs should recognize the crucial role of culture and its profound impact on ensuring business success in today’s competitive environment. It is imperative for international businesses to understand the national culture of the country they enter for a global business. Understanding culture could enable QSRs to provide NLMs that meet their needs and expectations. For example, in individualist societies such as the UK, QSRs could make use of media campaigns for increasing social influence on nutrition and calorie information. This will also enable QSRs to meet the needs of the national culture in relation to nutrition and calorie information.

## 6. Conclusions

The current research examined an extended model of TPB, which included three antecedents of behavioural intention (ATT, SNs, PBC) and health consciousness, to better understand consumers’ intentions to buy NLM items for healthy food choices in QSRs. The research compared the extended model between two countries with different cultures (the KSA and the UK) using a structural model of SMART PLS V4 and a multi-group analysis. The results of the structural model confirmed the extended model in both countries, except for the influence of SNs on the intention to buy NLM items among UK consumers and the influence of PBC on the intention to buy NLM items among KSA consumers. This means that consumers’ buying intentions of NLMs in QSRs in the UK are less likely to be affected by their friends and peers, mainly because they are an individualist society. On the other hand, KSA consumers are less likely to control their behaviour, mainly because of time or money. The research showed that health consciousness significantly influences intentions of KSA and UK consumers to buy NLM items, which was found to significantly impact the intention to recommend NLM items to others. The current research adds to academic literature by extending the TPB framework to better understand NLM buying intentions and behaviours. It also highlighted the primary role of culture in QSRs, especially in relation to buying intentions and behaviours towards NLM items, for making healthy food choices. The research was undertaken in two different countries using a self-reporting measure. Future research studies could consider other countries with a deeper analysis of their culture and its relationship with their buying intentions and behaviours.

## Figures and Tables

**Table 1:** The psychometric properties of the employed scale.

		UK					Saudi Arabia				
Construct	Item	Loading (CFA)	α	AVE	Composite Reliability	Variance Inflation Factor (VIF)	Loading (CFA)	α	AVE	Composite Reliability	Variance Inflation Factor (VIF)
Attitude towards Behaviour	Attitude1	0.855	0.895	0.898	0.761	1.417	0.760	0.838	0.854	0.673	1.595
	Attitude2	0.899					0.873				
	Attitude3	0.866					0.840				
	Attitude4	0.869					0.804				
Subjective Norms	SN1	0.904	0.854	0.934	0.765	1.491	0.954	0.944	0.945	0.899	1.607
	SN2	0.855					0.949				
	SN3	0.864					0.941				
Perceived Behavioural Control	PBC1	0.948	0.955	0.955	0.881	1.612	0.840	0.877	0.917	0.726	1.462
	PBC2	0.946					0.822				
	PBC3	0.929					0.874				
	PBC4	0.933					0.871				
Health Consciousness	Health_1	0.913	0.880	0.982	0.794	1.401	0.887	0.780	0.777	0.699	1.569
	Health_2	0.869					0.876				
	Health_3	0.890					0.737				
Buying Intention	Visit_1	0.890	0.849	0.855	0.770		0.866	0.836	0.839	0.753	
	Visit_1	0.918					0.895				
	Visit_1	0.821					0.843				
Recommendation Intention	Recomend_1	0.901	0.851	0.852	0.773		0.898	0.851	0.851	0.772	
	Recomend_1	0.919					0.917				
	Recomend_1	0.813					0.818				

Source: Authors.

**Table 2:** Cross loadings.

	UK						KSA					
Items	Attitude	Visit	Health.	PBC	Recommend	SN	Attitude	Visit	Health.	PBC	Recommend	SN
Attitude1	**0.855**	0.531	0.406	0.478	0.512	0.395	**0.760**	0.435	0.495	0.336	0.527	0.458
Attitude2	**0.899**	0.540	0.350	0.356	0.562	0.367	**0.873**	0.574	0.489	0.364	0.578	0.485
Attitude3	**0.866**	0.590	0.363	0.420	0.599	0.294	**0.840**	0.530	0.386	0.280	0.490	0.293
Attitude4	**0.869**	0.497	0.360	0.373	0.497	0.361	**0.804**	0.420	0.446	0.299	0.411	0.303
Helth_Consc.1	0.310	0.331	**0.913**	0.366	0.368	0.341	0.455	0.408	**0.887**	0.366	0.414	0.419
Helth_Consc.2	0.239	0.305	**0.869**	0.340	0.367	0.325	0.434	0.404	**0.876**	0.331	0.403	0.364
Helth_Consc.3	0.494	0.555	**0.890**	0.449	0.530	0.412	0.481	0.427	**0.737**	0.297	0.418	0.335
PBC1	0.415	0.575	0.442	**0.948**	0.596	0.434	0.369	0.466	0.376	**0.840**	0.457	0.485
PBC2	0.485	0.544	0.421	**0.946**	0.560	0.518	0.272	0.230	0.301	**0.822**	0.311	0.386
PBC3	0.434	0.566	0.421	**0.929**	0.610	0.490	0.326	0.327	0.352	**0.874**	0.404	0.467
PBC4	0.420	0.564	0.392	**0.933**	0.564	0.511	0.330	0.346	0.305	**0.871**	0.357	0.415
Recommend1	0.594	0.677	0.462	0.529	**0.901**	0.422	0.583	0.622	0.422	0.397	**0.898**	0.551
Recommend2	0.575	0.700	0.441	0.533	**0.919**	0.406	0.578	0.652	0.455	0.412	**0.917**	0.525
Recommend3	0.476	0.675	0.408	0.575	**0.813**	0.365	0.461	0.649	0.425	0.409	**0.818**	0.562
SN1	0.443	0.481	0.404	0.490	0.458	**0.904**	0.443	0.535	0.467	0.490	0.598	**0.954**
SN2	0.234	0.246	0.342	0.396	0.311	**0.855**	0.443	0.502	0.392	0.501	0.560	**0.949**
SN3	0.321	0.343	0.329	0.455	0.377	**0.864**	0.452	0.522	0.412	0.498	0.610	**0.941**
Visit1	0.571	**0.890**	0.448	0.512	0.681	0.369	0.579	**0.866**	0.431	0.353	0.616	0.460
Visit2	0.564	**0.918**	0.435	0.589	0.718	0.386	0.509	**0.895**	0.450	0.390	0.669	0.531
Visit3	0.497	**0.821**	0.382	0.471	0.650	0.392	0.485	**0.843**	0.413	0.365	0.616	0.434

Note: Bold items are cross loading for construct variables.

**Table 3:** Discriminant validity (UK data set).

	Fornell and Larker						HTMT Results					
	1	2	3	4	5	6	1	2	3	4	5	6
	**0.872**											
1-Attitude	0.621	**0.877**					0.709					
2-Buying intention	0.424	0.481	**0.891**				0.436	0.511				
3-Health consciousness	0.467	0.599	0.447	**0.939**			0.505	0.663	0.467			
4-Perceived behaviour control	0.625	0.779	0.498	0.621	**0.879**		0.712	0.917	0.543	0.689		
5-Recommendation intention	0.404	0.435	0.415	0.520	0.453	**0.875**	0.435	0.478	0.456	0.564	0.510	
6-Subjective norms	0.872											

Note: Bold values are the square root of all AVE values.

**Table 4:** Discriminant validity (KSA data set).

	Fornell and Larker						HTMT Results					
	1	2	3	4	5	6	1	2	3	4	5	6
	**0.821**											
1-Attitude	0.604	**0.868**					0.713					
2-Buying intention	0.550	0.497	**0.836**				0.682	0.613				
3-Health consciousness	0.390	0.425	0.398	**0.852**			0.443	0.467	0.471			
4-Perceived behaviour control	0.615	0.731	0.495	0.463	**0.879**		0.724	0.865	0.605	0.517		
5-Recommendation intention	0.471	0.548	0.448	0.523	0.622	**0.948**	0.527	0.615	0.521	0.563	0.694	
6-Subjective norms	0.821											

Note: Bold values are the square root of all AVE values.

**Table 5:** Hypotheses testing results.

	KSA				UK			
Hypotheses	β	*t*	*p*	Results	β	*t*	*p*	Results
**H1:** Attitude towards behaviour → Buying intention	0.367	7.530	0.000	Supported	0.385	8.184	0.000	Supported
**H2:** Subjective norms → Buying intention	0.267	5.385	0.000	Supported	0.046	1.226	0.220	Not Supported
**H3:** Perceived behaviour control → Buying intention	0.086	1.281	0.223	Not Supported	0.327	6.908	0.000	Supported
**H4:** Health consciousness → Buying intention	0.141	3.084	0.002	Supported	0.153	3.753	0.000	Supported
**H5:** Buying intention → Recommendation intention	0.731	20.746	0.000	Supported	0.779	24.832	0.000	Supported
**Specific Indirect Effects**								
Attitude towards behaviour → Buying intention → Recommendation intention	0.269	7.143	0.000	Supported	0.300	7.639	0.000	Supported
Subjective norms → Buying intention → Recommendation intention	0.195	4.847	0.000	Supported	0.036	1.222	0.222	Not Supported
Perceived behaviour control → Buying intention → Recommendation intention	0.063	2.272	0.023	Supported	0.255	6.408	0.000	Supported
Health consciousness → Buying intention → Recommendation intention	0.103	3.058	0.002	Supported	0.119	3.713	0.000	Supported

**Table 6:** Multi-group analysis.

Hypotheses	Difference (UK–KSA)	2-Tailed (UK vs. KSA) *p* Value
Attitude towards behaviour → Buying intention	0.017	0.797
Subjective norms → Buying intention	−0.221	0.000
Perceived behaviour control → Buying intention	0.241	0.000
Health consciousness → Buying intention	0.012	0.836
Buying intention → Recommendation intention	0.048	0.302
Attitude towards behaviour → Buying intention → Recommendation intention	0.031	0.563
Subjective norms → Buying intention → Recommendation intention	−0.160	0.001
Perceived behaviour control → Buying intention → Recommendation intention	0.192	0.000
Health consciousness → Buying intention → Recommendation intention	0.016	0.721

## Data Availability

Data is available upon request from researchers who meet the eligibility criteria. Kindly contact the first author privately through e-mail.

## Appendix A. The Study Measures

**Item**	**Item**	**Source**
**Attitude towards Behaviour**		Ajzen [15] and Shin et al. [19]
**Attitude1**	Using nutrition labelling for health food choices is an advantageous action	
**Attitude2**	Using nutrition labelling for health food choices is a wise action.	
**Attitude3**	Using nutrition labelling for health food choices is a pleasant action.	
**Attitude4**	Using nutrition labelling for health food choices is an attractive action.	
**Subjective Norms**		Ajzen [15] and Shin et al. [19]
SN1	People who are important to me think I should choose a nutrition-labelled item for health food choice when eating out.	
SN2	Most people who are important to me would want me to choose a nutrition-labelled menu item for health food choice when eating out.	
SN3	People whose opinions I value would prefer me to choose a nutrition-labelled item for health food choice when eating out.	
**Perceived Behavioural Control**		Ajzen [15] and Shin et al. [19]
PBC1	I am confident that if I want, I can choose a nutrition-labelled item for health food choice when eating out.	
PBC2	I am capable of choosing a nutrition-labelled item for health food choice when eating out.	
PBC3	I have enough resources (money) to choose a nutrition-labelled item for health food choice when eating out.	
PBC4	I have enough time to choose a nutrition-labelled item for health food choice when eating out.	
**Health Consciousness**		Shin et al. [20]
**Helth_Consc.1**	I think of myself as a health conscious consumer.	
**Helth_Consc.2**	I think often about health issues.	
**Helth_Consc.3**	I am planning to buy X when I dine out in the future.	
**Buying Intention**		Ajzen [15] and Shin et al. [19]
**Recommend1**	I am planning to choose a nutrition-labelled menu for health food choice item when eating out in the future.	
**Recommend2**	I intend to choose a nutrition-labelled menu for health food choice item when eating out in the future.	
**Recommend3**	I will expend effort on choosing a nutrition-labelled menu for health food choice menu item when eating out in the future.	
**Recommendation Intention**		Ajzen [15] and Shin et al. [19]
**Visit1**	I am planning to recommend a restaurant featuring nutrition labelling for health food choice when someone asks me to eat out in the future.	
**Visit2**	I intend to recommend a restaurant featuring nutrition labelling for health food choice when someone asks me to eating out in the future.	
**Visit3**	I will expend effort on persuading everybody who asked me to eat out to visit a restaurant featuring nutrition labelling for health food choice in the future.	
